# Safety of transanal ileal pouch-anal anastomosis for ulcerative colitis: a retrospective observational cohort study

**DOI:** 10.1186/s13037-021-00306-5

**Published:** 2021-09-18

**Authors:** Aina Lask, Matthias Biebl, Luca Dittrich, Andreas Fischer, Andreas Adler, Frank Tacke, Felix Aigner, Rosa Schmuck, Sascha Chopra, Michael Knoop, Johann Pratschke, Safak Gül-Klein

**Affiliations:** 1 Department of Surgery, Campus Charité Mitte and Campus Virchow-Klinikum, Charité – Universitätsmedizin Berlin, Augustenburger Platz 1, 13353 Berlin, Germany; 2 Department of Hepatology and Gastroenterology, Charité – Universitätsmedizin Berlin, Freie Universität Berlin and Humboldt Universität zu Berlin , Berlin, Germany; 3 Department of Surgery, Barmherzige Brüder Krankenhaus Graz, Graz, Austria

**Keywords:** Transanal ileal pouch-anal anastomosis, Pouchitis, Ulcerative colitis, Anastomotic leakage

## Abstract

**Background:**

Colectomy with transanal ileal pouch-anal anastomosis (taIPAA) is a surgical technique that can be used to treat benign colorectal disease. Ulcerative colitis is the most frequent inflammatory bowel disease (IBD) and although pharmacological therapy has improved, colectomy rates reach up to 15%. The objective of this study was to determine anastomotic leakage rates and treatment after taIPAA as well as short- and long-term pouch function.

**Methods:**

We conducted a retrospective analysis of a prospective database of all patients undergoing taIPAA at an academic tertiary referral center in Germany, between 01/03/2015 and 31/08/2019. Patients with indications other than ulcerative colitis or with adjuvant chemotherapy following colectomy for colorectal carcinoma were excluded for short- and long-term follow up due to diverging postoperative care yet considered for evaluation of anastomotic leakage.

**Results:**

A total of 22 patients undergoing taIPAA during the study time-window were included in analysis. Median age at the time of surgery was 32 ± 12.5 (14–54) years. Two patients developed an anastomotic leakage at 11 days (early anastomotic leakage) and 9 months (late anastomotic leakage) after surgery, respectively. In both patients, pouches could be preserved with a multimodal approach. Twenty patients out of 22 met the inclusion criteria for short and long term follow-up. Data on short-term pouch function could be obtained in 14 patients and showed satisfactory pouch function with only four patients reporting intermittent incontinence at a median stool frequency of 9–10 times per day. In the long-term we observed an inflammation or “pouchitis” in 11 patients and a pouch failure in one patient.

**Conclusion:**

Postoperative complication rates in patients with benign colorectal disease remain an area of concern for surgical patient safety. In this pilot study on 22 selected patients, taIPAA was associated with two patients developing anastomotic leakage. Future large-scale validation studies are required to determine the safety and feasibility of taIPAA in patients with ulcerative colitis.

## Background

Ulcerative colitis is the most common inflammatory bowel disease (IBD) and exceeds the incidence and prevalence of Crohn's disease in most countries around the world [1]. Over the past few decades, pharmacologic therapy has improved, limiting the need for surgery to cases of refractory or steroid-dependent disease, colorectal cancer or surgical emergencies such as toxic megacolon, perforation or life-threatening hemorrhages [2]. Throughout the course of the disease, medical therapy refractory ulcerative colitis, which requires surgical treatment, leads to reconstructive surgery rates by restoring continuity of up to 15% [3]. In this regard, the use of minimally invasive ileal pouch-anal anastomosis (IPAA) is the favored technique [4, 5].

Transanal total mesorectal excision was established for sphincter-preserving rectal resection due to rectal carcinoma, providing better access and overview especially in obese patients or male individuals with naturally narrow pelvises [6, 7]. Subsequently, transanal minimally invasive surgery has also been implemented in operations for transanal IPAA (taIPAA) and, as already proven in various studies, also provides satisfactory surgical results and comparable complication rates to the pure transabdominal approach [8–10]. Anastomotic leakage is a common complication in colorectal surgery with significant postoperative morbidity [11]. Irrespective of the presence of benign or malignant disease as a surgical indication, anastomotic leakage results in chronic inflammation, fistulae or stenosis, reduced quality of life and eventually anastomotic failure with permanent stoma [11–13]. Additionally, in colorectal carcinoma, anastomotic leakage is associated with reduced disease-free and overall survival [14, 15]. Besides anastomotic leakage, pouchitis is the most frequent complication of IPAA significantly impairing pouch function and quality of life [16, 17]. Anastomotic leakage in association to pouch complications might be of relevant impact for pouch failure after IPAA [9, 18].

The multimodal treatment procedures of anastomotic leakage management include—depending on the extent of anastomotic leakage—antibiotic therapy, radiological or endoscopic interventions as well as reoperation with the intend to preserve continuity, as recently shown by different groups for anastomotic leakage for rectal cancer and IBD [19–21].

Therefore, the aim of our retrospective study was to analyze patients after taIPAA due to benign colorectal disease regarding incidence and course of anastomotic leakage as well as episodes of pouchitis and associated hospital readmissions.

## Methods

### Study design

All consecutive patients subjected to a taIPAA in our department were analyzed retrospectively. All patients eligible for laparoscopic resection were offered the transanal approach. Those who chose taIPAA were prospectively recruited and gave written informed consent for the international LOREC® registry. The study was performed in accordance with the Declaration of Helsinki and its amendments and an authorization has been granted by the Charité Ethics Committee (Reg. No. 711/16). For clinical data such as sex, date of birth, preoperative data (therapy, body-mass-index (BMI), extent of inflammation), operative data (operation time) and postoperative course as well as short- and long-term follow-up data (complications according to Clavien-Dindo classification; readmissions), patient data were collected anonymously.

In our retrospective single-center observational cohort study we hypothesized, that taIPAA is a safe procedure for restorative proctocolectomy in patients suffering from ulcerative colitis and provides satisfactory short- and long-term outcome.

**Fig. 1:**
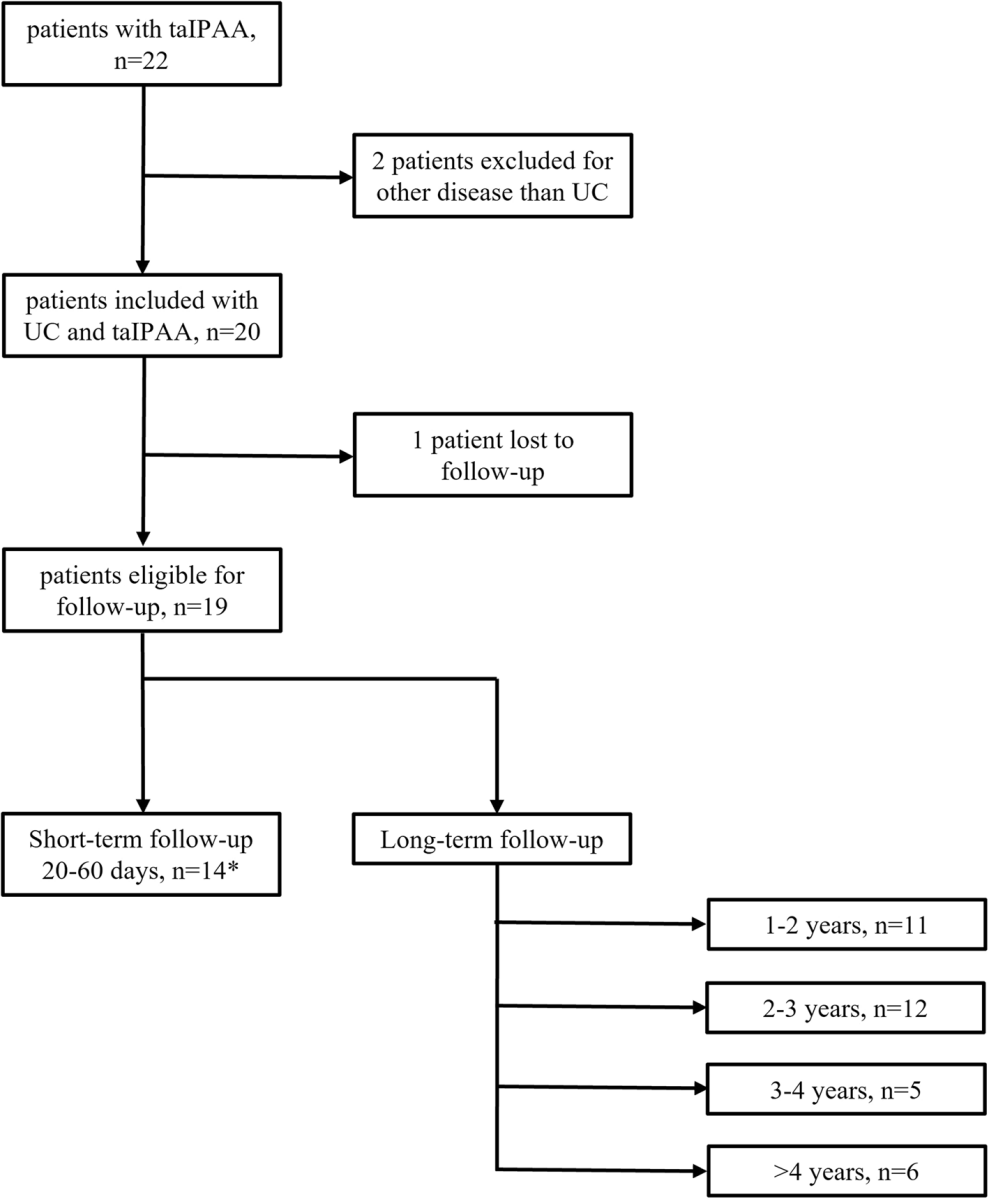
Patient cohort, * Five patients rejected routine postoperative follow up at our department of surgery within 20 to 60 days due to good clinical status in ambulatory control

From 01/03/2015 to 31/08/2019, taIPAA, performed in the context of a two- or three-staged restorative proctocolectomy, was applied in 22 patients suffering benign colorectal disease at our Department of Surgery, Campus Virchow Klinikum and Campus Charité Mitte at Charité—Universitätsmedizin Berlin. For the analysis of anastomotic leakage, we included all patients who received taIPAA. To evaluate short- and long-term postoperative outcome, we only considered taIPAA in patients with ulcerative colitis and hence excluded two patients. Of those, one suffered from familial adenomatosis polyposis and the other one was diagnosed with ulcerative colitis but excluded due to additional colorectal carcinoma with hyperthermic intraperitoneal chemotherapy prior to pouch formation and adjuvant chemotherapy after colectomy and therefore diverging postoperative care (Table 1 and Fig. 1).

**Table 1:** Patient characteristics and operative details

**Patients**	**n = 22**
Sex (male)	14
Median age (years, ± SD, range)	32 ± 12.5 (14–72)
Mean BMI (kg/m^2^, ± SD, range)	22.8 ± 3.7 (17–32.8)
ASA Score	
I	4
II	16
III	2
Pre-existing health conditions	
Smoker	0
Diabetes	2
Previous abdominal operation	5
Median time from diagnosis of ulcerative colitis to operation (months, ± SD, range)	36 ± 104.5 (1–408)
Three staged procedure	19
Two staged procedure	3
Mean operation time taIPAA (minutes, ± SD, range)	362 ± 163 (134–905)
Anastomosis	
Stapled	8
Handsewn	14
Cufflength (cm from pectinate line, median and range)	1 (0–3)

*BMI* Body Mass Index, *ASA* American society of Anaesthesiologists, *taIPAA* Transanal ileal pouch-anal anastomosis

Relevant data (clinical course, laboratory chemical parameters, hospital stay, readmissions) for our patient cohort were analyzed retrospectively. Postoperative complications were defined according to Clavien-Dindo with special emphasis on complications > II.

Occurrence of early (within the first 30 days) and late (31 to 90 days or later) anastomotic leakage was the primary endpoint. Anastomotic leakage is defined as dehiscence of the anastomotic circumference leading to a communication between the intra- and extraluminal compartments. In our study, diagnosis of anastomotic leakage was made when dehiscence of the anastomotic circumference was confirmed via endoscopy. Secondary endpoints included pouch complications (pouchitis, fistula, stenosis). These endpoints were assessed and evaluated at regular follow-ups at our Departments of Surgery or Gastroenterology. One patient did not receive ileostomy closure at our surgical department, hence data on short- and long-term follow up could not be obtained.

### Surgical technique

The whole operation procedure for ulcerative colitis was performed either as a two- or three-staged procedure with an interval of three to six months between procedures. The first operation comprised subtotal colectomy and terminal ileostomy, the second operation included an ileal proctectomy following the prior colectomy and pouch-anal anastomosis with diverting loop-ileostomy.

All patients were operated in a simultaneous transanal and abdominal technique. After excision and closure of terminal ileostomy followed the insertion of a single port and placement of the capnoperitoneum. With an additional 5 mm trocar in the left mid-abdomen the entire small intestine was mobilized and adhesions were removed up to the duodenum. Through the port, the J-pouch was created starting from the apex of the mesenteric vessel axis. The staple suture on the distal limb was sewn over and the apex of the pouch came to rest loosely below the symphysis. Following the pouch confection, mesorectal mobilization was carried out next. Simultaneously, after rinsing the rectal stump with NaCl-Iodide solution, a tobacco-pouch suture was placed 3 cm above the pectinate line to close the rectum. Subsequently, the rectum was incised 2 cm above the pectinate line and the single-incision-laparoscopic-surgery port was inserted and fixed to the perianal skin. Placement of the capnosubperitoneum was followed by the preparation of the mesorectal layer. After successful dissection using the rendez-vous method, the peritoneal cavity was reached and the rectum was retrieved via the port site. Ileal pouch-anal anastomosis was either stapled or handsewn followed by creation of a diverting loop-ileostomy. Perioperative antibiotic prophylaxis using cefuroxime and metronidazol was given to all patients.

The last operation consisted of ileostomy closure. Beforehand, an endoscopy of the pouch was performed to exclude severe inflammation (pouchitis) or anastomotic leakage. No further diagnostic measures (as e.g. CT-scan or rectal contrast application) were taken. Ileostomy closure only took place after constating absence of inflammation and inconspicuous anastomosis region.

In two patients, colectomy as well as pouch confection were performed simultaneously due to one patients excellent inflammation remission and the other patients diagnosis of non-inflammatory familiar adenomatosis polyposis. One patient in our cohort did not undergo loop ileostomy due to severe adhesions. Diverting loop ileostomy is standard at our institution, but may be considered optional in patients at very low risk for anastomotic leakage and/ or increased risk for complications. All operations for pouch confection were conducted in a hybrid technique including minimally invasive transabdominal approach and combined transanal approach.

### Data collection

Data was collected in a prospective database for patients receiving taIPAA and analyzed retrospectively for perioperative complications and postoperative clinical course. Subsequent data were analyzed: demographics (age, gender), body-mass-index (BMI), American society of Anaesthesiologists (ASA) score, comorbidities, preoperative course of disease, operative details (Table 1), postoperative morbidity and mortality, complications with special focus on anastomotic leakage (time of diagnosis, treatment approach, treatment duration) (Table 2), short-term (first 60 days after ileostomy closure) (Table 3), and long-term (1 to 5 years after ileostomy closure) pouch function (Table 4), pouchitis (clinical symptoms, confirmation via endoscopy), fistula, stenosis and pouch failure (Table 5).

**Table 2:** Anastomotic leakage and course of treatment

**Patient No**	**3**	**4**
Endo-SPONGE® therapy (frequency)	1	2 / 4
Length of treatment (days)	3	5 / 12
Transanal suture	-	Yes
Reopening of ileostomy	-	Yes
Preserved anastomosis	Yes	Yes
Anastomotic leakage related mortality	-	-

**Table 3:** Short-term pouch function (20–60 post operation)

**Patients**	**n = 14**
Stool frequency (n = 11)	
2–5	1
6–8	3
9–10	2
10–20	5
> 20	0
Incontinence	
None	10
Intermittent	4
Permanent	0
Pouchitis	0
Fistula	0
Stenosis	0
Pouch failure	0

**Table 4:** Long-term pouch function

	**1–2 years**	**2–3 years**	**3–4 years**	** > 4 years**
n	11	12	5	6
anastomotic leakage	1	1	0	0
Pouchitis	5	5	2	0
Fistula	1	3	1	1
Stenosis	0	1	0	0
Pouch failure	1	1	1	0

**Table 5:** Overview to pouchitis related characteristics

Patient No	1	5	6	8	11	12	13	14	16	18	20
Age	34	39	30	41	35	48	33	33	17	36	59
BMI	26.3	22.2	22	20.2	29.6	32.8	21.5	20.4	22.5	17	30.9
Cardiovascular disease	-	-	-	-	-	-	-	-	-	-	-
Diabetes	-	-	-	-	-	-	-	-	-	-	-
First diagnosed (days p.o.)	586	1231	309	625	819	298	237	91	459	360	210
Extraintestinal manifestation											
- Arthritis	Yes	-	-	-	-	Yes	-	-	-	-	-
- Dermatitis	Yes	-	-	-	-	Yes	-	-	-	-	-
- Primary sclerosing cholangitis	-	-	-	-	Yes	-	Yes	-	-	-	-
- Other	-	-	-	-	-	-	-	Yes^a^	-	-	-
Stenosis	-	-	-	-	-	-	-	-	-	-	Yes
Fistula	Yes	-	-	-	-	Yes	-	-	-	-	Yes
Treatment											
- Antibiotics	Yes	Yes	Yes	Yes	Yes	Yes	-	Yes	-	-	-
- Steroids (topical)	-	Yes	Yes	-	-	-	-	-	-	-	-
- Steroids (systemic)	-	-	Yes	-	-	-	-	-	-	-	-
- 5-aminosalicylic acid (topical)	-	Yes	Yes	-	-	Yes	-	-	-	Yes	-
- Biologicals	-	-	Yes	-	-	Yes	Yes	-	Yes	-	-

^a^idiopathic portal vein hypertension, *BMI* Body mass index

### Data analysis

Data analysis was performed using Microsoft Excel. Continuous variables were reported as mean or median values with range. Categorical variables were quantified using frequencies and percentage. Since the study population is too small to perform valid statistical testing, results are presented in a merely descriptive manner. Follow-up period started at the day of last operation.

## Results

### Patient characteristics and operative details

Gender distribution presented more male patients (male 14 patients; female 8 patients) with median age of 32 ± 12.5 (14–54) years at the time of taIPAA. The median time from first diagnosis of ulcerative colitis to subtotal colectomy was 36 ± 104.5 (1 – 408) months. Nineteen patients received a three-staged procedure as described earlier. Two patients were operated in a two-staged procedure with colectomy and pouch-formation in the same operation. Another patient did not receive diverting loop-ileostomy due to multiple adhesions in the second procedure. Detailed information on patients’ demographics and clinical features as well as on the operation are shown in Table 1.

### Anastomotic leakage and postoperative clinical course

Complications according to Clavien-Dindo classification > II took part over all three operation procedures, in total five patients. We observed one patient with severe complications for colectomy, three for pouch formation and three for ileostomy closure. After colectomy one patient developed a partial tear of terminal ileostomy which had to be addressed in the operating room. After pouch formation complications comprised gastrointestinal bleeding due to Mallory-Weiss lesions under therapeutic anticoagulation, high output stoma with acute kidney failure and consecutive temporary dialysis and early anastomotic leakage. After ileostomy reversal we saw two perforations located proximal to the ileo-ileal anastomosis and one anastomotic leakage of the ileo-ileal anastomosis.

Two patients of our cohort developed anastomotic leakage of ileal pouch-anal anastomosis. One patient was diagnosed with early anastomotic leakage on the 13th postoperative day. The second patient showed a late anastomotic leakage 19 months after diverting loop-ileostomy closure. Both patients were primarily treated with endosponge therapy and pouches could be preserved. The early anastomotic leakage required only one session of endosponge replacement, while late anastomotic leakage needed a multimodal approach including multiple endosponge changes.

The patient with late anastomotic leakage first received two cycles of endosponge therapy followed by a transanal suture completing the multimodal therapy procedure. Recurrence of anastomotic leakage 51 days after the aforementioned transanal suture was again treated with four cycles of endosponge therapy and re-operation for performing a diverting loop-ileostomy after the first cycle (Table 2). Definitive ileostomy closure could be achieved 380 days after last treatment for anastomotic leakage.

### Pouch function in the short- and long-term

Clinical data of pouch function in the short-term was obtained for 14 patients (Table 3) with a median follow-up time of 39.5 ± 9.2 (29–59) days. None of the patients displayed any signs of pouchitis, fistula or stenosis. Pouch function was sufficient, as no permanent incontinence was observed. Intermittent incontinence, comprising stress induced incontinence and seepage due to liquid stool, was documented in four patients. Stool frequencies were reported for 11 patients, with one patient at a frequency of 2–5 times per day, three patients at 6–8 times per day and two patients at 9–10 times per day. Five patients recorded stool frequencies of 11–20 times per day.

In the long-term follow-up, comprising 1 to 5 years, 11 patients out of 19 patients developed pouchitis, at a mean of 475 ± 327.3 (91–1231) days after ileostomy closure (Table 5). Six patients were diagnosed within the first year after ileostomy closure.

Additionally, three patients developed a fistula, all in direct coincidence to ileal-pouch-anal anastomosis. Out of those fistulas two were blind fistulas and one was a pouch-vaginal fistula with intermittent vaginal defecation.

The eight remaining patients had no endoscopic signs of pouchitis, while one presented a blind, asymptomatic fistula during routine postoperative endoscopy. One patient with pouchitis presented additional stenosis of the ileal pouch-anal anastomosis and was treated six times via ambulatory endoscopic dilatation. In 8 patients out of those 11 patients with pouchitis, a successful treatment was achieved with pharmacological therapy, comprising mainly antibiotics, topical and systemic steroids and in severe cases biologicals (Table 5). Meanwhile only 3 patients out of the pouchitis group still showed signs of pouchitis at last follow up. Here one patient was recommended for pouch removal and terminal ileostomy due to severe pouchitis with multiple fistulae. To date, the patient is in regular internal and surgical control and declines pouch removal.

## Discussion

Transanal surgery has been studied mainly in transanal total mesorectal excision as therapy for rectal carcinoma and provides comparable oncological outcomes, yet better pathologic resection status, shorter operation time, lower conversion rate and lower overall postoperative complication rate in comparison to laparoscopic total mesorectal excision [12, 22–26]. Although the transanal approach for oncological surgery has been well researched since it was first performed for total mesorectal excision in 2010 [7] and offers improved surgical access to the otherwise often challenging, narrow pelvis [6], it is still not as widely established for benign indications such as ulcerative colitis. Currently, taIPAA is reported to provide comparable functional results and postoperative morbidity and mortality to the transabdominal approach with shorter operation time and conversion rate, but still study populations are relatively small and randomized controlled trials have not been conducted so far [8, 9, 27].

In our study we observed complications r(Clavien-Dindo > II) in three patients for pouch formation, which is comparable to results reported recently for transabdominal IPAA [17] and even slightly lower than in two studies concerning taIPAA [8, 9]. Nevertheless, these studies also showed higher complication rates for transabdominal IPAA resulting in no significant difference between both approaches. Anastomotic leakage occurred in two patients in our study. These results are consistent with findings for IPAA before implementation of the transanal approach [18, 28], and similar anastomotic leakage rates have also been shown for taIPAA [8, 9]. Chandrasinghe et al*.* even reported a trend for lower anastomotic leakage rates in taIPAA compared to transabdominal IPAA, which did not reach statistical significance [8] and could not be verified in our study. Course of treatment for anastomotic leakage after taIPAA has not yet been reported in detail.

In our study, only one early anastomotic leakage and one late anastomotic leakage was observed. Treatment was aimed at preserving the anastomosis and therefore founded on endosponge therapy. While early anastomotic leakages would be treated only with endosponge therapy, late anastomotic leakages required a multimodal approach consisting of endosponge therapy, transanal suture and reopening of the diverting loop-ileostomy. Our therapeutic approach relies on our experiences after transanal total mesorectal excision for rectal carcinoma, where we developed a differentiated therapy strategy for anastomotic leakage [20]. Endosponge therapy for anastomotic leakage after IPAA has not yet been widely researched, but the few studies existing show non-inferiority in comparison to conventional treatment and high rates of pouch preservation [21, 29].

In our study, we did not observe any pouch failure due to anastomotic leakage with an overall pouch failure in one patient, which is also consistent with earlier findings [8, 17]. The only detected patient with pouch failure had an underlying severe pouchitis which was therapy refractory to pharmalogical treatment and therefore recommended for pouch removal and terminal ileostomy.

Pouchitis is one of the most frequent complications after IPAA [28, 30] and associated with significant morbidity and reduced quality of life [31, 32]. Pouchitis rates after IPAA reach up to 60% with increasing incidence during postoperative follow-up [16, 30, 33] and may result in chronic pouch dysfunction and in severe cases pouch failure [34]. The overall pouchitis rate was 11 patients, with half of the patients developing symptoms within one year after the last operation. Furthermore, pharmacological treatment was successful in 8 patients resulting in a good functional outcome in long-term follow-up. There are still only few studies on treatment for acute and chronic pouchitis leaving therapeutic strategies with highly variable outcomes and lacking sufficient scientific evidence [35, 36].

In order to improve therapy for pouchitis more research is needed. In the only study comparing long-term results of taIPAA and transabdominal IPAA, no significant difference was found neither between pouchitis rates nor health related quality of life including sexual function [8]. Long-term follow-up for transabdominal IPAA shows over 90% of patients being satisfied with the operative result, despite minor complications such as nocturnal seepage or higher daily stool frequencies [17, 37, 38].

Our study is limited by its retrospective design without option to rule out potential bias, especially selection bias. The restricted number of patients does not suffice for statistical evaluation of our results. However, the monocentric design of our study ensures that the same experienced colorectal surgeons’ team performed all procedures, resulting in excellent comparability of postoperative outcomes.

## Conclusion

In our study, anastomotic leakage rates, postoperative complications as well as short- and long-term outcome of taIPAA were comparable to previously published results for only tranabdominal surgery. Still, postoperative complications remain an area of concern in colorectal surgery for benign colorectal disease, especially ulcerative colitis. Since taIPAA was introduced only a few years ago, studies for long-term outcomes and evaluation of larger patient cohorts are necessary to fully understand possible advantages or downsides of this surgical approach.

## Data Availability

The datasets used and/or analyzed during the current study are available from the corresponding author on reasonable request.

## Abbreviations

ASA: American society of Anaesthesiologists
BMI: Body mass index
IBD: Inflammatory bowel disease
talPAA: Transanal ileal pouch-anal anastomosis
